# Quantification of the incidence and financial implications of stock theft in Eastern Cape Province, South Africa

**DOI:** 10.1007/s11250-025-04634-x

**Published:** 2025-09-30

**Authors:** Kanya Ndzungu, Yusuf Bitrus Ngoshe, Ishmael Festus Jaja

**Affiliations:** 1 Department of Livestock and Pasture Science, University of Fort Hare, Alice, South Africa; 2 Department of Production Animal Studies, Faculty of Veterinary Science, University of Pretoria, Private Bag X04, Onderstepoort, 0110 South Africa; 3 Department of Agriculture and Animal Health, University of South Africa, Roodepoort, Johannesburg, South Africa

**Keywords:** Agricultural sustainability, Cattle, Eastern Cape Province, Farm losses, Livestock theft

## Abstract

Stock theft poses a significant and persistent challenge to the agricultural sector in South Africa. This widespread issue has severe consequences for farmers, including financial losses, emotional distress, and the inability to maintain sustainable agricultural practices. The objective of the study was to quantify the incidence and financial implications of stock theft in Eastern Cape Province (ECP), South Africa. The retrospective data (2018 to 2024) of stock theft was obtained from the provincial police stock theft unit, captured in Excel, transformed, and analysed using R Studio 4.2.2 (version 2022) for statistical analysis. The Alfred Nzo district, with a total of 6427 (21.5%) reported cases, experienced the highest number of cases of stock theft reported amongst the eight districts of the ECP in the period from 2018 to 2024 financial year. Sheep (OR = 1.03; *P* < 0.01) and goat (Odds Ratio = 1.00; *P* > 0.40) had a higher likelihood of being stolen, and with no recoveries after being stolen (Odds Ratio = 0.99; *P* > 0.93) and (Odds Ratio = 1.01; *P* > 0.119). In the current study, cattle amounted to a loss of 399 million rands (21,904,418.00 USD), with 30,816 cattle being stolen. The impact of stock theft has far-reaching ramifications for the country’s economy and the sustainability of current farming ventures. Information obtained from this study could assist stakeholders in being empowered to make informed decisions, allocate resources effectively, and develop targeted interventions to combat stock theft.

## Introduction 

The Eastern Cape Province is one of South Africa’s key agricultural regions, particularly renowned for its significant livestock population (Clack 2013; Maluleke and Dlamini 2019). Additionally, the National Agricultural Census (NAC) has provided that the province is home to around 3.9 million cattle, 10.2 million sheep, and 2.4 million goats, making it a leader in livestock production (Young et al. 2023). The ECP, compared to other provinces, has the largest sheep production (10.2 million), followed by KwaZulu-Natal (2.4 million), Free State (1.4 million), and Northern Cape (1.2 million) (Zantsi and Greyling 2021; Malete 2023; Stats 2022). Furthermore, the province’s various livestock species also include game animals, horses, and donkeys, which contribute to its reputation as an important agricultural centre.

Livestock farming is the livelihood for most farmers and a vital component of nutritional and food security (Eeswaran et al. 2022). Additionally, this industry contributes significantly to the creation of earnings, food security and nutrition, the supply of draught power, and the availability of manure for approximately 85% of Africa’s rural population, primarily dependent on crop production and livestock (Birhanu et al. 2023). Recently, South Africa’s livestock owners have found it challenging to make a livelihood due to the ongoing problem of stock theft, which impacts food security (Maluleke 2022). The primary cause of the overall increase in stock theft numbers is the demand for meat to supply the growing population of Africa, including South Africa (Thornton 2010a, b).

The rising livestock numbers in the Eastern Cape Province (ECP) have been accompanied by a worrying increase in incidents of stock theft (Aiyzhy et al. 2021; Birhanu et al. 2023). This correlation points to a worrying trend: despite its detrimental impact on agriculture and the livelihoods of the rural population, the issue of stock theft continues to be inadequately addressed in South Africa (Aerni-Flessner et al. 2021; Lombard and Bahta 2019). The lack of severe measures to combat this crime means that it is not treated with the severity it deserves, creating an environment conducive to the continued exploitation of farmers. Hence, this study aims to quantify the incidence of stock theft and its financial implications in Eastern Cape Province, South Africa.

## Materials and methods

### Ethical certificate

The ethical clearance certificate was obtained and released by the University of Fort Hare Research Ethics Committee (UFHREC) in 2022 with the project number **JAJ031SNDZ01** before the process of data collection, and the ethical clearance certificate is **REC-270710-028-RA Level 01**. Approval was obtained from each participant prior to the commencement of the interview. Participation in the survey was voluntary, and participants were informed of their right to withdraw from the survey at any stage.

### Research design and study area

**Fig. 1:**
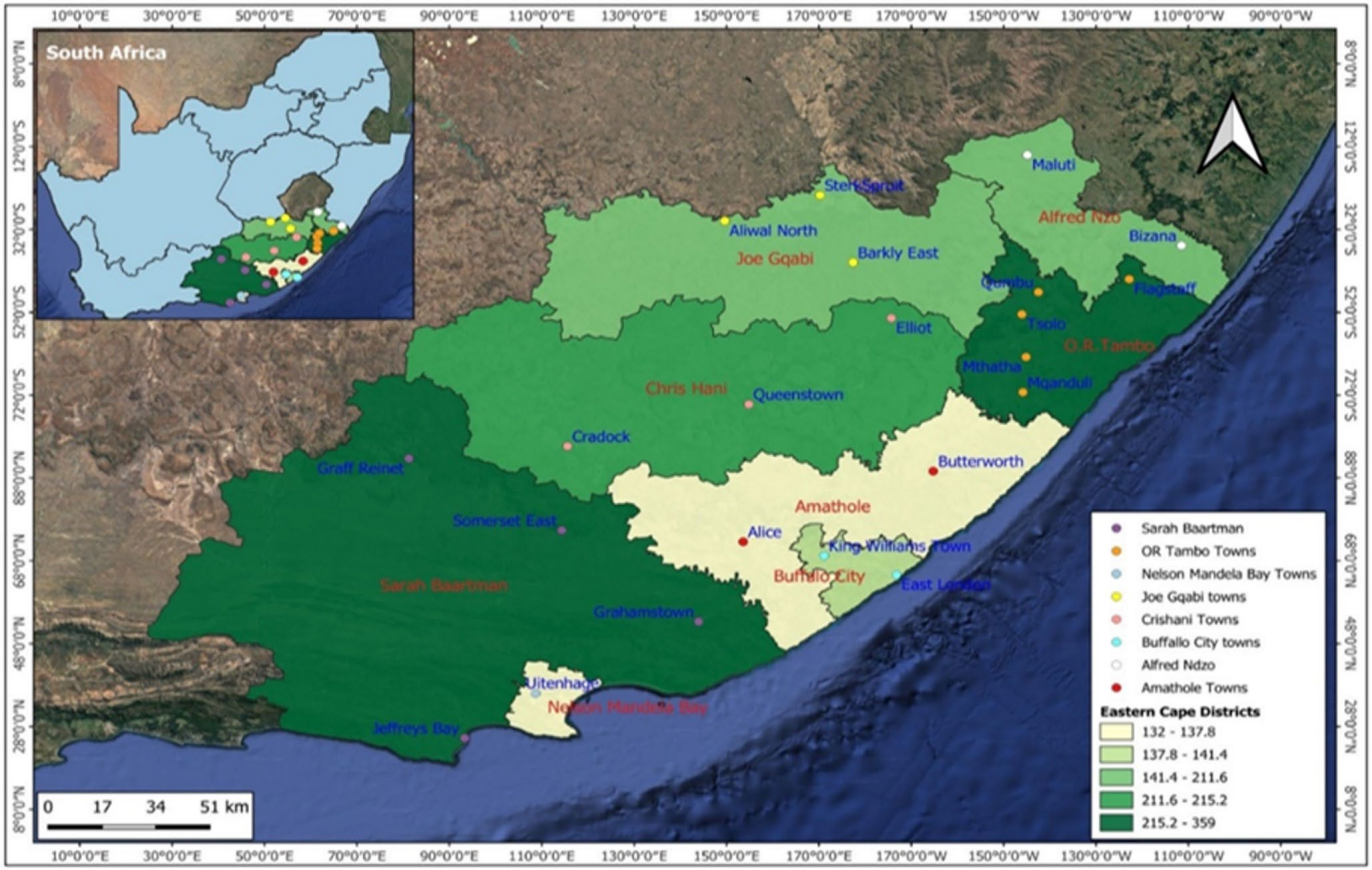
The distribution of stock theft units across the district’s municipality of Eastern Cape Province, South Africa

This study used a cross-sectional research design (Bryman 2012) to investigate the incidence and financial implications of stock theft in ECP, South Africa. Retrospective data was collected from the Provincial Police Head Office in Zwelitsha, King William’s Town. The data collected was for all the ECP districts: Alfred Nzo, Amathole, Buffalo Metropolitan City, Chris Hani, Joe Gqabi, Nelson Mandela Bay, OR Tambo, and Sarah Baartman. The South African Police Service (SAPS) has strategically divided these districts into 21 stock theft units (STUs), and these STUs are specifically and explicitly designed to prevent stock theft around ECP (Fig. 1). Additionally, the study focused on domesticated animals, including cattle, goats, pigs, horses, donkeys, chickens, ostriches, and game animals.

The Eastern Cape Province is renowned for having a large population of livestock, for example, about 3.9 million cattle, 10.2 million sheep, 2.4 million goats, 484,368 pigs, and 6.4 million chickens, which unfortunately correlates with a higher incidence of stock theft (Oduniyi et al. 2020; Stats 2022). It also has about 165,738 household farming cattle, 151,276 household sheep, 166,994 household farming goats, 139,735 household farming pigs, and 263,790 farming chickens (Stats 2022). The abundance of animals in this region creates an environment conducive to stock theft, making it a significant concern for law enforcement and livestock owners.

### Data collection

The stock theft data was collected from the provincial office of the SAPS for six years, starting from 2018 to 2024 (April-March) financial year, according to the SAPS calendar. The data in the head office is stored in the security system software called Crime Administration System (CAS). For analysis purposes, they use specialised software tools such as crimesStar, crimeView, or ArcGIS. The data was extracted and transferred to Microsoft Excel 2016 to accommodate the process of coding and removing unwanted variables to facilitate statistical analysis. Additionally, to calculate the percentage of recovered, stolen, and not recovered animals, the following formulas:

$$\begin{aligned}&\text{Recovered\:of\:each\:species\:in\:each\:district}\cr&\quad\left(\frac{Number\:of\:recovered}{Total\:number\:of\:stolen\:species}\times100\%\right)\end{aligned}$$

$$\begin{aligned}&\text{Stolen\:of\:each\:species\:in\:each\:district}\cr&\quad\left(\frac{number\:of\:stolen\:animals}{\begin{aligned}&Total\:number\:of\:stolen\cr&\quad\:animals\:in\:each\:species\:\end{aligned}}\times\:100\%\right),\end{aligned}$$

$$\begin{aligned}&\text{Not\:recovered\:animals\:in\:each\:species}\cr&\quad\left(\frac{\begin{aligned}&Total\:number\:of\cr&\quad\:unrecovered\:animals\end{aligned}}{\begin{aligned}&Total\:number\:of\:reported\cr&\quad\:stolen\:animals\:\end{aligned}}\times\:100\%\right)\end{aligned}$$

### Financial implications

Six farmers were called in each district to estimate financial loss over the period of 6 years (2018–2024) in stolen animals, asking for the market price of each animal considered in the study (Table 1). The market price for each animal was obtained by dividing the prices provided by commercial and communal farmers in the study area. Furthermore, to convert the rand to the dollar, we applied an exchange ratio, 1 United States Dollar (USD):18.10 ZAR, using a currency conversion website (www.xe.com/currencyconverter/convert/?Amount=1%26From=ZAR%26To=USD).

**Table 1:** The average market prices of each animal considered in the study

Species									
	Cattle	Sheep	Goat	Horse	Donkey	Ostriches	Pig	Game	Poultry
Average Price (ZAR)	R12 876.35	R1 675.10	R2021.90	R7 500.00	R5000.00	R115.00	R2500.00	R118.00	R130.00
Average Price (USD)	1287.64 USD	92.54 USD	111.71 USD	414.36 USD	276.24 USD	6.35 USD	138.12 USD	6.51 USD	6.51 USD

In the study, animals’ age was not taken into consideration. However, farmers were asked to provide a single market price for each animal species, regardless of age, so this means separate prices for female and male animals were asked of farmers, as their market values may differ (Table 1).

### Statistical analysis

The study applied R Studio 4.2.2 (version 2022) for statistical analysis. The p-value was used to determine associations between the stolen animals (Cattle, sheep, goat, horse, donkey, ostriches, pig, game, and poultry) and the districts of the ECP. Additionally, a logistic regression analysis was conducted to investigate the relationship between livestock theft and several key predictor variables: the number of reported cases (No. Cases), the number of animals stolen (No. Stolen), and the number recovered (No. Recovered). Separate logistic regression models were created for each species to evaluate species-specific dynamics. The outcome variable was binary, coded as 1 if an event occurred (e.g., the recovery of stolen livestock) and 0 if it did not. The predictor variables were treated as independent, and the models estimated odds ratios, p-values, and 95% confidence intervals. This approach allowed for the quantification of the likelihood of theft or recovery events in relation to species type and other factors, such as geographic district, thereby providing insights into the patterns of livestock theft and recovery across different species.

## Results

### Reported cases of stock theft and recovered stolen animals from 2018 to 2024 financial year

Alfred Nzo district (21.5%) and Sarah Baartman district (20.8%) had the highest reported stock theft cases compared to other districts. Also, Alfred Nzo had the highest reported cases of cattle theft (33.3%) and horse theft (57.6%), then OR Tambo district comes second with the reported cases of cattle theft (21.1%) and Horse theft (23.7%) compared to other districts (Table 2). Additionally, Chris Hani had the highest reported cases of sheep theft (22.8%) compared to other districts. Furthermore, 39.2% of goats were highly reported in the Sarah Baartman district compared to other districts. Poultry (28.6%) and Pig (38.4%) had the highest reported stock theft cases in Nelson Mandela Bay compared to other districts. Among the types of species, sheep (42.2%) had the most reported stock theft cases (Table 2).

**Table 2:** Reported cases of stock theft from 2018–2019 to 2023–2024 financial year

District	Species									
	Cattle (%)	Sheep (%)	Goat (%)	Horse (%)	Donkey (%)	Ostriches (%)	Pigs (%)	Game (%)	Poultry (%)	Total (%)
Amathole	1343 (15.4)	1201 (9.5)	847 (14)	119 (10.4)	44 (22.4)	0 (0)	98 (16.8)	18 (10.6)	89 (23.8)	3759 (12.6)
Alfred Nzo	2910 (33.3)	1844 (14.6)	865 (14.3)	658 (57.6)	126 (64.3)	0 (0)	0 (0)	0 (0)	24 (6.4)	6427 (21.5)
Buffalo City Metropolitan	28 (0.3)	11 (0.1)	20 (0.3)	1 (0.1)	0 (0)	0 (0)	0 (0)	1 (0.6)	6 (1.6)	67 (0.22)
Chris Hani	1124 (12.9)	2866 (22.8)	626 (10.4)	67 (5.9)	2 (1)	3 (12)	77 (13.2)	34 (20)	47 (12.6)	4846 (16.2)
Joe Gqabi	345 (3.9)	1372 (10.8)	176 (2.9)	14 (1.2)	2 (1)	18 (72)	49 (8.4)	3 (1.8)	25 (6.7)	2004 (6.7)
Nelson Mandela Bay	418 (4.8)	537 (4.3)	728 (12.1)	12 (1)	11 (5.6)	3 (12)	224 (38.4)	27 (15.9)	105 (28.1)	2065 (6.9)
OR Tambo	1841 (21.1)	1927 (15.3)	409 (6.8)	271 (23.7)	9 (4.6)	1 (4)	11 (1.9)	0 (0)	10 (2.7)	4479 (15)
Sarah Baartman	724 (8.3)	2837 (22.5)	2367 (39.2)	1 (0.1)	2 (1)	0 (0)	124 (21.3)	87 (51.2)	68 (18.2)	6210 (20.8)
**Total**	8733 (29.2)	12,595 (42.2)	6038 (20.2)	1143 (3.8)	196 (0.7)	25 (0.1)	583 (2)	170 (0.7)	374 (1.3)	29,857 (100)

Sarah Baartman (37.1%) and Amathole (26.9%) districts had the highest successful recovery of stolen animals, while the least recovered stolen animals were in the Joe Gqabi (11.8%) and Chris Hani (9.6%) districts. Also, the most successful district in recovering stolen cattle was Buffalo Metropolitan (26%), followed by Alfred Nzo (25.6%) and Amathole (24.9%). 3821 (30.1%) and 980 (28.6%) sheep stolen were successfully recovered in Amathole and Sarah Baartman, respectively. Horses and Donkeys recovered the most in Joe Gqabi (70.7%) and Sarah Baartman (66.7%). Furthermore, A total of 66 (58.4%) and 62 (33.9%) of game and poultry were most recovered in Sarah Baartman, respectively (Table 3).

**Table 3:** Recovered stolen animals from 2018 to 2024 financial year

District	Species									
	Cattle (%)	Sheep (%)	Goat (%)	Horse (%)	Donkey (%)	Ostriches (%)	Pigs (%)	Game (%)	Poultry (%)	**Total (%)**
Amathole	1075 (24.9)	3822 (30.1)	1422 (27.4)	38 (22)	17 (27)	1 (0)	7 (4.8)	0 (0)	24 (2)	6406 (26.9)
Alfred Nzo	4418 (25.6)	5962 (14.8)	2245 (19.7)	408 (33.8)	61(31.9)	0 (0)	6 (0)	0 (0)	0 (0)	13,100 (18.6)
Buffalo metropolitan	13 (26)	2 (3.8)	38 (29.2)	0/0 (0)	0 (0)	0 (0)	0 (0)	2 (100)	0 (0)	55 (14.9)
Chris Hani	1038 (12.4)	2698 (9.2)	614 (8.1)	16 (15.2)	0 (0)	0 (0)	16 (7.8)	24 (15.3)	0 (0)	4406 (9.6)
Joe Gqabi	326 (23.8)	1492 (9.6)	354 (26.5)	29 (70.7)	0 (0)	0 (0)	6 (6.2)	0 (0)	0 (0)	2207 (11.8)
Nelson Mandela Bay	300 (18.1)	563 (19.5)	400 (12.5)	10 (52.6)	14 (50)	0 (0)	64 (11.7)	9 (23.7)	38 (1.8)	1398 (13.3)
OR Tambo	1224 (21.6)	6065 (16.4)	882 (20.1)	88 (22.7)	1 (5.6)	0 (0)	6 (7.4)	0 (0)	9 (13.6)	8275 (17.4)
Sarah Baartman	563 (56)	980 (28.6)	1035 (40.3)	0 (0)	2 (66.7)	0 (0)	73 (61.9)	66 (58.4)	62 (33.9)	2781 (37.1)
**Total**	8957 (22.6)	21,584 (15.3)	6990 (19.6)	589 (30.5)	95 (34.8)	1 (2.5)	178 (14.8)	101 (29)	133 (2.8)	38,628 (17.2)

NB: To find % in recovered from each species in districts, ($\:\frac{Number\:of\:recovered}{Total\:number\:of\:stolen\:species}\times100\%$) for each species

### The total estimation of stock theft reported cases stolen and recovered from the 2018–2024 financial year in the Eastern Cape Province

Sheep were the most stolen animals, totalling 119,404 (57.1%), resulting in a loss of 148 million rands (11,041,352.97 USD). Goats followed them, with 51,881 stolen (24.8%), causing an estimated loss of 104 million rands (5,790,695.00 USD). Cattle ranked third, with 30,816 stolen (14.7%), but due to their higher market value, the total loss amounted to 399 million rands (21,904,418.00 USD). Furthermore, the total value of stolen animals is 715 million rands (39,494,283.60 USD), with 93.1% being lost and not recovered (Table 4).

**Table 4:** Unrecovered stolen animals from 2018/2019 to 2023/2024 financial year including the total estimation loss

District	Species									
	Cattle (%)	Sheep (%)	Goat (%)	Horse (%)	Donkey (%)	Ostriches (%)	Pigs (%)	Game (%)	Poultry (%)	**Total (%)**
Amathole	3244 (10.7)	8871 (7.4)	3769 (7.3)	135 (10)	46 (31.9)	1 (2.4)	136 (14.7)	2 (0.93)	1203 (26.5)	17 407 (8.3)
Alfred Nzo	12,843 (42.5)	34,223 (28.7)	9147 (17.6)	798 (59.3)	61 (42.4)	0 (0)	0 (0)	0 (0)	257 (5.7)	57 329 (27.4)
Buffalo metropolitan	37 (0.12)	50 (0.04)	92 (0.18)	2 (0.15)	0 (0)	0 (0)	16 (1.7)	2 (0.93)	118 (2.6)	317 (0.15)
Chris Hani	7416 (24.6)	26,491(22.2)	6975 (13.4)	89 (6.6)	3 (2.1)	18 (42.9)	137 (14.8)	133 (62.1)	394 (8.7)	41 656 (19.9)
Joe Gqabi	1041 (3.4)	14,116 (11.8)	983 (1.9)	12 (0.89)	2 (1.4)	19 (45.2)	33 (3.6)	0 (0)	258 (5.7)	16 464 (7.9)
Nelson Mandela Bay	1362 (4.4)	2318 (1.9)	2798 (5.4)	9 (0.67)	14 (9.7)	3 (7.1)	485 (52.3)	30 (14)	2133 (47)	9152 (4.4)
OR Tambo	4430 (14.4)	30,884 (25.9)	3503 (6.8)	300 (22.3)	17 (11.8)	1 (2.4)	75 (8.1)	0 (0)	57 (1.3)	39 267 (18.8)
Sarah Baartman	443 (1.4)	2451(2.1)	24,534 (47.3)	1 (0.07)	1 (0.69)	0 (0)	45 (4.9)	47 (22)	121 (2.7)	27,643 (13.2)
**Total**	30,816 (14.7%)	119 404 (57.1%)	51,881 (24.8%)	1346 (0.6)	144 (0.1)	42(0.02)	927 (0.4)	214 (0.1)	4541 (2.2)	209 235
**Loss in** *** R***	399 m	148 m	104 m	10 m	720 000	4830	2,3 m	25 200	590 330	715 m
**Loss in** *** USD***	21 904 418.00	11 041 352.97	5 790 695.00	557 274.29	38 999.98	266.59	127 932.95	1 372.98	31 970.80	39 494 283.6
**% loss in stolen animals**	78.2	62.8	68.9	69.6	52.7	95.2	76.9	61.5	97.1	93.1

NB: To find the % Not recovered animals, ($\:\frac{Total\:number\:of\:unrecovered\:stolen\:animals}{Total\:number\:of\:reported\:stolen\:animals\:}\times\:100\%$), *R* means South African Rand and *USD* means United states dollar

The total number of stolen animals (384,730) is significantly higher than that of recovered animals (59,237). There is a wide range of values for each type of livestock, indicating significant variation in the data. Additionally, the number of livestock stolen varies widely, with some incidents involving large numbers of animals. The confidence intervals provide a range of values for the mean number of livestock stolen (Table 5).

**Table 5:** The total estimation of stock theft reported cases stolen and recovered from 2018–2024 financial year in the Eastern Cape Province

	Total	Mean	Std. Dev.	Max	Std. Err.	[95% Conf.	Interval]
Cattle	46,649	39.86	81.10	2525	3022.96	40717.86	52580.14
Sheep	196,860	159.56	237.67	2879	8487.75	180206.80	213513.2
Goats	50,555	42.44	53.20	407	1922.30	46783.37	54326.63
Horses	1842	1.58	4.44	47	161.94	1524.26	2159.74
Donkey	345	0.04	1.47	15	41.10	264.37	425.63
Ostriches	49	0.04	0.52	18	20.08	9.60	88.40
Pigs	1728	1.35	3.86	63	141.40	1450.56	2005.44
Game	619	0.45	7.18	273	278.96	71.70	1166.33
Poultry	5860	4. 75	21.25	320	743.57	4401.08	7318.922
Number of cases	54 887	34.65	40.05	425	1.01	32.71	36.66
Number of Stolen	384 730	242.89	298.39	3383	7.51	228.39	257.84
Number of Recoveries	59 237	37.44	58.87	667	1.48	34.54	40.35

### Unrecovered stolen animal and estimated financial loss from 2018 to 2024 financial year

Alfred Nzo (27.4%) and Chris Hani (19.9%) had the highest rate of unrecovered stolen animals compared to other districts. Cattle (42.5%) and sheep (28.7%) were the most unrecovered stolen animals under Alfred Nzo, followed by Chris Hani with cattle (24.6%) and sheep (22.2%), and OR Tambo with cattle (14.4%) and sheep (25.9%) unrecovered of stolen animals. 24,534 (47.3%) goats were reported as unrecovered stolen animals in Sarah Baartman, followed by 9147 (17.6%) in Alfred Nzo. Additionally, Alfred had the highest number of horses (59.3%) and donkeys (42.4%) compared to other districts. Furthermore, Nelson Mandela Bay also had the highest number of pigs (52.3%) and poultry (47%) unrecovered after being stolen compared to other districts (Table 4).

### Logistic regression analysis by species showing the total number of cases stolen and recovered

Sheep (Odds Ratio = 1.03; *P* < 0.01) had a higher likelihood of being stolen and with no recovery after being stolen (OR = 0.99; *P* > 0.93). Additionally, it is more likely to recover cattle (Odds Ratio = 1.02; *P* < 0.01) when stolen as compared to sheep (Odds Ratio = 0.99; *P* > 0.93) and goat (Odds Ratio = 1.01; *P* > 0.119). Furthermore, the analysis reveals varying likelihoods of theft and recovery across different species, with sheep, goats, pigs, and poultry showing significant associations (Table 6). Moreover, game, donkey, and ostriches show no significant associations (*P* > 0.05) in the number of cases reported and several recoveries (Table 6). The results indicate minimal associations for all outcomes in cattle. The most consistent finding is a small (2%) but statistically significant increase in the odds of recovery. However, the clinical or practical significance of these results seems limited due to the effect sizes.

**Table 6:** Logistic regression analysis by species showing total number of cases, stolen and recovered animals

Species	Observation	Odds ratio	*P*-value	95% conf.	Interval
**Cattle**					
No. Cases No. stolen No. recover	1 532	0.99 1.02 1.02	0.04 0.72 < 0.01	0.99 1.01 1.01	1.00 1.02 1.04
**Sheep**					
No. Cases No. Stolen No. Recovered	1 551	1.19 1.03 0.99	< 0.01 0.01 0.93	1.07 1.01 0.63	1.32 1.06 2.89
**Goat**					
No. Cases No. Stolen No. Recovered	1 464	1.12 1.00 1.01	< 0.01 0.401 0.119	1.09 1.00 1.00	1.2 1.00 1.02
**Horse**					
No. Cases No. Stolen No. Recovered	1 431	1.00 1.00 1.01	0.062 < 0.01 < 0.01	1.00 1.00 1.01	1.01 1.00 0.21
**Donkey**					
No. Cases No. Stolen No. Recovered	1521	1.00 1.00 1.00	0.205 < 0.01 0.486	0.99 1.00 1.00	1.00 1.00 0.12
**Ostriches**					
No Cases No Stolen No Recovered	1 568	1.00 1.00 1.00	0.339 0.211 0.327	1.00 1.00 0.98	1.01 1.00 1.01
**Pigs**					
No Cases No Stolen No Recovered	1 482	1.01 1.00 1.00	< 0.01 < 0.01 0.03	1.00 1.00 0.99	1.01 1.00 1.00
**Game**					
No Cases No Stolen No Recovered	1540	1.00 1.00 1.00	0.24 < 0.01 0.44	1.00 1.00 0.99	1.01 1.00 1.00
**Poultry**					
No Cases No Stolen No Recovered	1 485	1.00 1.00 1.00	0.01 0.22 0.01	1.00 1.00 0.99	1.01 1.00 1.00

## Discussions

In this study, the Alfred Nzo district experienced the highest number of reported stock theft cases among the eight Eastern Cape Province districts (ECP) from the 2018–2024 financial year. The study findings in Alfred Nzo were similar to those obtained in Limpopo (Giyani) province and Maluti police station (Maluleke et al. 2014). Additionally, Alfred Nzo, Or Tambo, and Amathole reported a high proportion of stock theft. This high incidence in Alfred Nzo may be attributed to its geographical location. It shares international borders with Lesotho and internal borders with OR Tambo, Amathole, and Joe Gqabi districts, facilitating easy cross-border movement of stolen animals (Aerni-Flessner et al. 2021; Clack 2018; Khoabane and Black 2012).

The Chris Hani and Sarah Baartman districts reported the highest cases of stock theft involving sheep and goats, respectively. Chris Hani district is noted for its significant sheep farming communities, while Sarah Baartman district is a major producer of goats in the ECP. The prominence of sheep and goat farming in these districts makes them prime targets for livestock theft (Malusi et al. 2021). Nelson Mandela Bay, known for its high pig and poultry meat production, also experienced significant theft cases involving pigs and poultry. This is consistent with previous studies by Simbizi et al. (2021, 2022), which reported a high prevalence of pig and poultry farming in the region, contrasting with the findings of Dyeyi and Zenda (2022).

The Amathole district had the highest recovery rate of stolen animals compared to Alfred Nzo and OR Tambo. Despite having the highest number of stolen animals, Alfred Nzo and OR Tambo had lower recovery rates, indicating a need for improved recovery strategies. The high recovery rate in Amathole can be attributed to effective community involvement, proactive measures, and strong collaboration among local farmers, police, and animal welfare organisations (Pasiwe et al. 2021). Community engagement is crucial for addressing livestock theft issues effectively.

Sarah Baartman also showed a high cattle, sheep, and goat recovery rate. This may be due to its strong agricultural focus and the relatively smaller district size, which facilitates better monitoring and control of theft syndicates. However, the overall recovery rate was less than a quarter of the stolen animals recovered, highlighting the urgent need for enhanced theft prevention strategies.

Alfred Nzo reported the highest incidence of unrecovered stolen animals, followed by Chris Hani and OR Tambo. The larger populations in these districts might contribute to higher theft opportunities and lower recovery rates (Stats 2022). Additionally, Chris Hani had a high incidence of unrecovered game animals, likely due to its rich biodiversity, which attracts poachers (Denner et al. 2024).

Sheep and goat were the most unrecovered animals compared to cattle. Sheep and goats are more difficult to recover after being stolen for various reasons than cattle. Their smaller size makes them easier to conceal and often lack distinctive markings or branding, making identification and tracking more difficult (Lombard 2016; Lombard and Bahta 2019). Additionally, sheep and goats are generally less valuable than cattle, which can lead to lower priority and fewer resources dedicated to recovery efforts (Makhatho et al. 2024).

The Alfred Nzo and OR Tambo districts have experienced the highest rates of horse theft. This trend can be attributed to horses’ significant role in agriculture and horse breeding in these districts (Marume et al. 2013). Notably, horse breeding is a prominent activity in Alfred Nzo and OR Tambo, setting them apart from other districts in the ECP (Mhlauli and Ezeuduji 2022; Ngumbela 2023). Additionally, in Alfred Nzo and OR Tambo, horse riding competitions and events such as dressage and horse shows are popular recreational activities, and these districts host several horse-riding clubs and organisations.

Cattle, having the highest market value, accounted for a loss of 399 million rands, with a total of 30,816 being stolen. Although it might seem challenging for stock thieves to steal cattle due to their large size, the high demand for red meat and the promise of easy profit make it a tempting target (Makhatho et al. 2024). Stock thieves know that successfully stealing cattle will be profitable due to their market value. Additionally, sheep and goats had losses of 148 million rands (11,041,351.97 USD) and 104 million rands (5,790,695.00 USD), with a total of stolen animals estimated to be 119,404 and 51,881, respectively. They are also significant in ECP culture and traditions, often used in various traditional ceremonies (Boyazoglu et al. 2005; Landau and Provenza 2020). They are frequently used in funerals, marriages (*untsiki*), and initiation ceremonies (*umgidi*). They are also offered to ancestors to request guidance, blessings, and protection.

Stock theft has far-reaching negative consequences that impact various aspects, such as the economy, domestic market, export, and food security. A total of 715 million rands (USD 39 494 283.6) was lost due to stock theft in the ECP from the 2018/2019 to 2023/2024 financial year. The findings are consistent with those of Maluleke et al. (2016), who reported a total loss of 613 million rands from 01 April 2010 to 31 March 2011 financial year. However, for the whole of South Africa, for the current study, we focused on the ECP for 6 years.

The current study suggests that stock theft is a significant threat to the farming industry in the ECP, with an overall 93.1% of losses attributed to stolen animals in the past six years. These losses can be attributed to stock theft, which only highlights the severity of stock theft in the farming industry in the ECP(Maluleke et al. 2022; Pasiwe 2021). This reflects the findings ofClack (2015, 2018), which identified stock theft as a priority crime in the National Rural Safety Strategy of SAPS. Furthermore, increasing stock theft incidents have depleted farmers’ trust and confidence in the police’s ability to stop criminality. In some cases, it has led to self-help by farmers, leading to incidents of farm murders (DAFF 2018).

## Conclusions

In the present study, Alfred Nzo had the highest proportion of stolen animals, followed by Chris Hani and OR Tambo, pinpointing the severity of the stock theft problem in these districts. Additionally, it is important to note that stock thieves target the strong farming communities as the districts above. Sheep and goats are more likely to be stolen among other animals due to their ability to be easily concealed and often lack distinctive markings or branding.

The study highlighted that there is still so much research needed on stock theft because it affects not only farmers but also the economy of the country. Despite the widespread nature of stock theft in the ECP of South Africa, research on its financial implications remains scarce. The limited availability of studies on this critical issue hinders stakeholders’ understanding of the severity of stock theft’s economic impact. It is recommended that future studies adopt a multidisciplinary approach, incorporating economic analysis, criminology, and rural development perspectives to provide a more comprehensive understanding of stock theft and its broader consequences.

## Data Availability

The data is available upon request.
